# Oiling-out effect improves the efficiency of extracting aroma compounds from edible oil

**DOI:** 10.1038/s41538-020-00079-8

**Published:** 2020-11-04

**Authors:** Daisuke Suzuki, Yuko Sato, Hiroshi Kamasaka, Takashi Kuriki, Hirotoshi Tamura

**Affiliations:** 1 Institute of Health Sciences, Ezaki Glico Co., Ltd., 4-6-5 Utajima, Nishiyodogawa-ku, Osaka, 555-8502 Japan; 2 The United Graduate School of Agricultural Sciences, Ehime University, 3-5-7 Tarumi, Matsuyama-shi, Ehime 790-8566 Japan; 3 The Graduate School of Agriculture, Kagawa University, 2393 Ikenobe, Miki-cho, Kagawa 761-0795 Japan

**Keywords:** Industry, Agriculture, Technology

## Abstract

Volatile compounds in foods are a significant factor that affects food intake and preference. However, volatile components in edible oils are poorly understood due to a strong matrix effect. In this study, we developed a method of extracting volatile compounds from extra virgin coconut oil (EVCO) by means of oiling-out assisted liquid-liquid extraction (OA-LLE). Consequently, 44 aroma compounds were isolated and identified from only 5 g of EVCO. Various aroma compounds were detected in addition to δ-lactones. The ratio of the natural abundance of the enantiomers of δ-lactones in EVCO was also revealed. Compared with the conventional methods of solvent assisted flavor evaporation (SAFE) and head-space solid-phase micro extraction (HS-SPME), OA-LLE was able to isolate a wide range and large number of volatile compounds from EVCO without leaving oil residues. Therefore, isolating aroma compounds from edible oil based on the oiling-out effect should provide an innovative extraction method.

## Introduction

Edible oil is one of the essential nutrients for human health and is a valuable primary material for many products. Therefore, many kinds of edible oil, e.g., olive oil, coconut oil, safflower oil, and lard, are produced and consumed worldwide. According to the OECD-FAO Agricultural Outlook 2019–2028, the global consumption of vegetable oils is over 200 million tonnes/year and is expected to continue to increase^1^ because demand is rising out of proportion to the world population.

It is well known that low molecular weight compounds affect the perception of food as flavor palatability^2–4^. In particular, volatile compounds in foods are a significant factor that affects food intake and preference. To understand the native profile of volatile compounds in edible oils, several methods to extract the volatile compounds have been developed so far.

Head-space solid-phase micro extraction (HS-SPME) introduced by Arthur and Pawliszyn in 1990 has been widely used to extract volatile compounds^5^. HS-SPME is a simple and solvent-free extraction method, and has been used to extract volatile compounds from various kinds of food including edible oils^6–9^. Solvent assisted flavor evaporation (SAFE) introduced by Engel et al. in 1999 has also been used to extract volatile compounds from edible oil^10–12^. SAFE can extract volatile compounds under mild conditions and can separate non-volatiles. In addition, an aromatic extract can be obtained to evaluate aroma characteristics. These methods have greatly contributed to the development of food science and the food industry. However, HS-SPME and SAFE are affected by the matrix effect of oil (triacylglycerols) during extraction processes for oil-enriched samples^10,13^. Some problems remain that need to be resolved, but there are few studies on how to extract volatile compounds from a matrix of oil. Indeed, the latest studies still use the HS-SPME or SAFE methods proposed in the 1990s for extracting volatile compounds from edible oils^8,9,12,14,15^. There have been significant developments in analytical instruments over many years, but extraction methods have become a bottleneck. To encourage and accelerate research on edible oils and fats, an efficient extraction method for volatiles from oil is desired.

Accordingly, we proposed an extraction method for extracting volatile compounds from dark chocolate (fat content, 35.3% w/w) based on the oiling-out effect^16^. Because the partition coefficient (log Pow) of long-chain triacylglycerols is very high, the triacylglycerols changed the distribution of the hexane and methanol bilayer in liquid-liquid extraction. We found that triacylglycerols are maintained in the hexane layer and the volatile compounds are collected in the methanol layer; this phenomenon was defined by a model study and named the “oiling-out effect”. The oiling-out assisted liquid-liquid extraction (OA-LLE) enabled us to extract 54 aroma compounds from only 5 g of dark chocolate. This method consisted of liquid-liquid extractions, and therefore, there are many advantages, e.g., less susceptible to the matrix effects of triacylglycerols, no heating process, small scale, easy to perform, and no need for expensive equipment. From these results, we hypothesized that OA-LLE can also be used to extract volatile compounds from edible oil. Edible oils consist of mostly triacylglycerols (around 100%), so edible oils can be regarded as the most difficult sample for extracting volatile compounds due to the strong matrix effect.

Coconut oil, which has a unique aroma, is nowadays widely used around the world. Early studies have shown that the key aroma compounds of coconut meat are δ-lactones, δ-octalactone and δ-decalactone^17,18^. However, there are few studies on volatile compounds in coconut oil^19–21^. Indeed, the enantiomers of δ-lactones contained in coconut oil have not been reported. Data on the enantiomer ratio of aroma compounds is important in helping to understand the aroma characteristics and the authenticity control of flavors^22^. However, the volatile composition of coconut oil is poorly understood due to a strong matrix effect.

In this study, OA-LLE was used to extract volatile compounds from only 5 g of extra virgin coconut oil (EVCO) as a typical model, and we evaluated the extraction efficiency of OA-LLE compared with HS-SPME and SAFE. In addition, the enantiomers of δ-lactones in EVCO were analyzed by gas chromatography-mass spectrometry (GC-MS) equipped with an enantioselective column, the results of which were reported. The method described in this paper should prove to be valuable in determining the volatile composition of edible oils, and should contribute to promoting research in food science as well as its associated industry.

## Results

### Extraction of volatile compounds in EVCO using OA-LLE

**Fig. 1:**
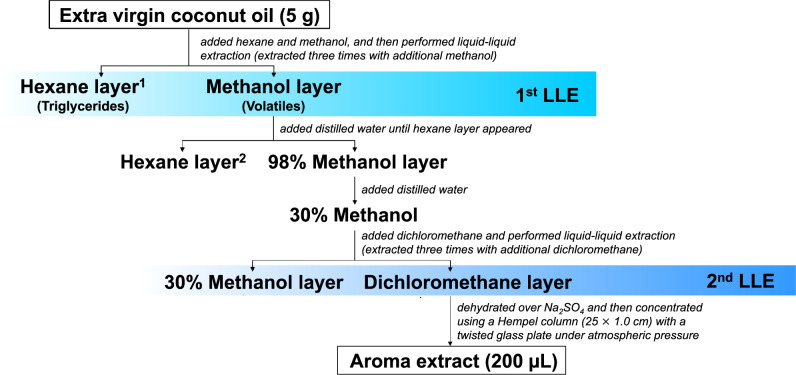
Procedure of the oiling-out assisted liquid–liquid extraction (OA-LLE) for extra virgin coconut oil (EVCO). OA-LLE consists of two small-scale liquid–liquid extractions.

Liquid-liquid extraction is a fundamental and important separation technique for a wide range of applications in scientific research and industries. Figure 1 presents the procedure of OA-LLE for EVCO. OA-LLE consists of two liquid-liquid extractions, hexane/methanol and 30% methanol solution/dichloromethane bilayers. At the first liquid-liquid extraction, the oil content in “hexane layer^1^” was 20.0% (w/v). From our previous study, it was considered that the oiling-out effect occurred^16^. After the OA-LLE, the hexane layers recovered 99.2 ± 0.6% (*n* = 3, mean ± standard deviation (SD), w/w) of the oil, indicating most of triacylglycerol was maintained in the hexane layer. To confirm the removed layers (“hexane layer^1^”, “hexane layer^2^”, and “30% methanol layer”) were odorless, three researchers sniffed a mouillette (smelling strip) dipped in these layers and sensorily judged that there was no odor in “hexane layer^1^” and “30% methanol layer”. There was a very slight odor in “hexane layer^2^”, perhaps because it did not contain enough oil for the oiling-out effect to occur. In contrast, the extract (concentrate of the “dichloromethane layer”) had a strong coconut-like aroma, and the typical aroma of coconut was recovered. These results indicate that most of the volatile compounds in EVCO were extracted by using OA-LLE. The extract was used for further analysis.

### Volatile compounds in EVCO

**Fig. 2:**
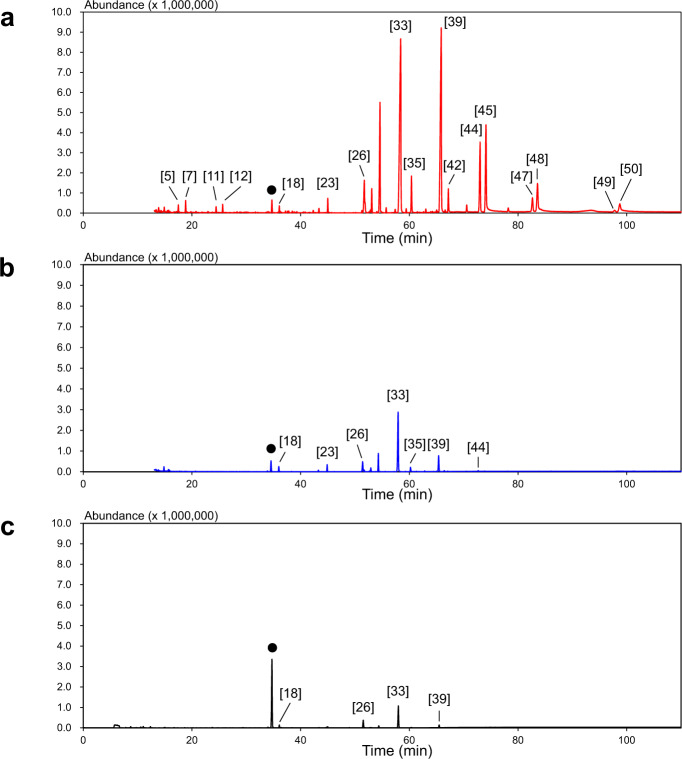
GC–MS chromatograms of the EVCO extracts from each extraction method. The chromatograms of OA-LLE, solvent assisted flavor evaporation (SAFE), and head-space solid-phase micro extraction (HS-SPME) are shown in **a**–**c**, respectively. Numbering refers to the volatile compounds listed in Table 1. Filled black circles (•) indicate internal standard peaks (cyclohexanol).

**Fig. 3:**
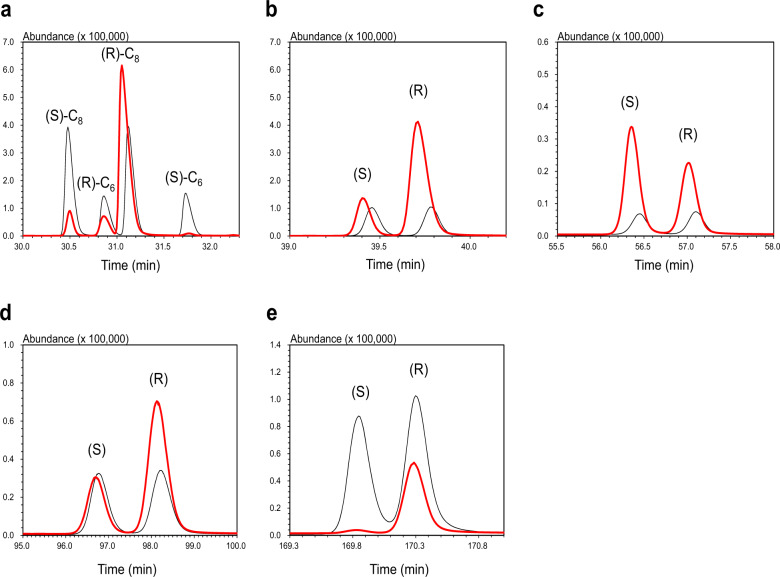
Enantioselective GC-MS chromatograms of δ-lactones. The chromatograms of the OA-LLE extract are drawn with a red and bold line, and a black line denote those of the standard solutions. The peaks of δ-hexalactone, δ-octalactone, δ-decalactone, δ-dodecalactone, δ-tetradecalactone, and δ-hexadecalactone are shown in **a**, **a**–**e**, respectively. Each % area of (R)- and (S)-enantiomers is shown in Table 3.

The GC-MS chromatograms and volatile compounds from OA-LLE, SAFE, and HS-SPME are shown in Fig. 2 and Table 1, respectively. In all, 50 volatile compounds were identified in EVCO using the three methods. With OA-LLE, 44 volatile compounds, comprising 8 acids, 13 alcohols, 3 aldehydes, 4 esters, 3 hydrocarbons, 7 ketones, and 6 lactones, were identified. Most of the volatile compounds detected in EVCO, such as nonanal and decanoic acid, were derived from triacylglycerols. δ-Lactones, δ-hexalactone (δ-C_6_), δ-octalactone (δ-C_8_), δ-decalactone (δ-C_10_), δ-dodecalactone (δ-C_12_), δ-tetradecalactone (δ-C_14_), and δ-hexadecalactone (δ-C_16_), were the major component of the volatiles in EVCO, and the content of these δ-lactones in the extract was 53.5 ± 6.2, 173.0 ± 14.6, 353.0 ± 17.5, 277.6 ± 10.7, 13.6 ± 0.9, and 2.1 ± 0.6 µg/200 µL (*n* = 3, mean ± SD), respectively. A large amount of δ-lactones was extracted. Applying available thresholds in oil or water, the odor activity value (OAV) (also odor unit) was calculated. The potential odorants (OAV > 1) detected in the OA-LLE extract are listed in Table 2. In total, 14 potential odorants were found. δ-Lactones have a chiral center in their compounds, so these compounds have enantiomers. As shown in Fig. 3 and Table 3, the predominance of (R)-enantiomers decreased with an increasing lactone chain length by δ-C_12_. δ-C_14_ and δ-C_16_ (R)-enantiomers increased up to 72.1 and 95.9%, respectively.

**Table 1:** Volatile compounds identified in EVCO.

No.	RI	Volatile compound	CAS	Log Pow^1^	Odor^2^	OA-LLE		SAFE		HS-SPME	Quantification^4^	Identification^5^
						Peak area^3^	µg/200 µL	Peak area	µg/200 µL	Peak area		
1	800	Octane	000111-65-9	3.9	Alkane, fruity, fusel, sweet	0.0	0.0	0.0	0.0	32.5	—	RI, MS, Std
2	930	Ethanol	000064-17-5	−0.1	Alcoholic, ethanol, pungent, sweet	0.0	0.0	0.0	0.0	354.5	—	RI, MS
3	998	Decane	000124-18-5	5.0	Alkane, fruity, fusel, sweet	0.0	0.0	0.0	0.0	167.7	—	RI, MS, Std
4	1036	Toluene	000108-88-3	2.7	Caramelized, ethereal, fruity, paint, pungent, rubber, solvent, synthetic	492.8	0.8	0.0	0.0	66.4	Std	RI, MS, Std
5	1049	3-Hexanone	000589-38-8	1.2	Ethereal, fresh, fruity, grape	2,419.7	5.6	0.0	0.0	0.0	B	RI, MS
6	1072	Hexanal	000066-25-1	1.8	Acorn, fatty, fishy, fruity, grassy, green, herbaceous, leafy, tallowy	0.0	0.0	0.0	0.0	65.0	—	RI, MS
7	1079	2-Hexanone	000591-78-6	1.4	Cinnamon, ethereal, fruity	3,692.7	8.5	0.0	0.0	0.0	Std	RI, MS, Std
8	1168	3-Penten-2-ol	001569-50-2	0.8	Green, vinyl	245.8	0.5	0.0	0.0	0.0	C	RI, MS
9	1173	2-Heptanone	000110-43-0	2.0	Cheese, cured ham, fruity, gaseous, gravy, nutty, soapy, toasted	0.0	0.0	0.0	0.0	143.1	—	RI, MS
10	1194	Dodecane	000112-40-3	6.1	Alkane, fusel	0.0	0.0	0.0	0.0	37.9	—	RI, MS, Std
11	1195	3-Hexanol	000623-37-0	1.7	Alcoholic, ethereal, medicinal	2,312.7	4.8	0.0	0.0	0.0	C	RI, MS
12	1220	2-Hexanol	000626-93-7	1.8	Fatty, fruity, winey	2,631.7	5.4	0.0	0.0	0.0	Std	RI, MS, Std
13	1285	1,2,4-Trimethylbenzene	000095-63-6	3.0	Herbaceous, plastic	345.0	0.5	0.0	0.0	0.0	A	RI, MS
14	1353	1-Hexanol	000111-27-3	2.0	Dry, floral, fruity, grassy, green, herbaceous, leafy, mild woody, resinous, sweet, toasty	225.0	0.4	0.0	0.0	0.0	C	RI, MS
15	1373	4-Ethyl-1,2-dimethylbenzene	000934-80-5	3.4	Green	61.3	0.1	0.0	0.0	0.0	A	RI, MS
16	1392	2-Nonanone	000821-55-6	3.1	Baked, earthy, fatty, fruity, green, hot milk, soapy	307.4	0.7	186.1	0.4	195.0	B	RI, MS
17	1396	Nonanal	000124-19-6	3.3	Chlorine, citrus, fatty, floral, fruity, gaseous, gravy, green, lavender, melon, soapy, sweet, tallowy, waxy	287.1	0.7	60.8	0.1	0.0	Std	RI, MS, Std
18	1438	Ethyl octanoate	000106-32-1	3.5	Anise, baked fruity, fatty, floral, fresh, fruity, green, leafy, mentholic, soapy, sweet, waxy	1,693.4	1.7	1,425.8	1.5	791.4	D	RI, MS
19	1446	Acetic acid	000064-19-7	−0.2	Acidic, pungent, sour, vinegar	327.6	2.2	0.0	0.0	160.6	Std	RI, MS, Std
20	1489	2-Ethylhexan-1-ol	000104-76-7	3.1	Citrus, green, mild, oily, rose, slightly floral rosy	469.5	0.9	60.8	0.1	67.4	C	RI, MS
21	1509	2,5-Hexanedione	000110-13-4	−0.3	Balsamic vinegar, mustard, pungent	422.4	1.0	0.0	0.0	0.0	Std	RI, MS, Std
22	1604	2-Undecanone	000112-12-9	4.1	Dusty, fresh, fruity, green, musty, orange	1,037.8	1.1	449.8	0.5	103.4	Std	RI, MS, Std
23	1644	Ethyl decanoate	000110-38-3	4.6	Fruity, grape, waxy	3,675.8	3.8	2,137.0	2.2	334.3	Std	RI, MS, Std
24	1720	2-Undecanol	001653-30-1	4.5	Fruity	122.6	0.1	0.0	0.0	0.0	E	RI, MS
25	1773	(E,Z)-2,4-Decadienal	025152-83-4	3.2	Deep-fried, fatty, geranium, metallic, tallowy	148.9	0.3	0.0	0.0	0.0	F	RI, MS
26	1813	δ-Hexalactone	000823-22-3	1.0	Fatty, herbaceous	14,303.5	53.5	4,240.7	16.0	3,800.8	Std	RI, MS, Std
27	1820	2-Tridecanone	000593-08-8	5.2	Herbaceous, spicy, waxy	1,985.0	2.0	599.8	0.6	62.4	G	RI, MS
28	1820	(E,E)-2,4-Decadienal	025152-84-5	3.2	Citrus, deep-fried, fatty, green, pungent, waxy	455.0	0.8	0.0	0.0	0.0	Std	RI, MS, Std
29	1844	Hexanoic acid	000142-62-1	1.9	Cheese, fatty, goat, pungent, rancid, sweaty	925.3	2.3	359.2	0.9	120.6	Std	RI, MS, Std
30	1851	Ethyl dodecanoate	000106-33-2	5.6	Leafy, mango, waxy	7,376.6	7.7	1,375.1	1.4	80.1	D	RI, MS
31	1925	2-Tridecanol	001653-31-2	5.6	Sweet fruity	1,350.8	1.2	89.4	0.1	0.0	E	RI, MS
32	1972	1-Dodecanol	000112-53-8	5.1	Fatty, waxy	1,020.6	0.9	0.0	0.0	0.0	E	RI, MS
33	2000	δ-Octalactone	000698-76-0	1.9	Peach, sweet	125,022.4	173.0	29,391.9	40.9	9,384.3	Std	RI, MS, Std
34	2031	2-Pentadecanone	002345-28-0	6.3	Floral, herbaceous, spicy	1,114.9	1.2	0.0	0.0	0.0	G	RI, MS
35	2060	Octanoic acid	000124-07-2	3.0	Cheese, fatty, fatty acid, fresh, mossy, sweaty	11,942.2	22.4	2,317.4	4.3	174.3	Std	RI, MS, Std
36	2124	2-Pentadecanol	001653-34-5	6.7	Floral	260.1	0.2	0.0	0.0	0.0	E	RI, MS
37	2156	2-Phenoxyethanol	000122-99-6	1.2	Fain floral-rose, floral	119.4	0.2	0.0	0.0	0.0	Std	RI, MS, Std
38	2169	Nonanoic acid	000112-05-0	3.5	Fatty, green, musty, sour, sweaty, waxy	305.7	0.4	0.0	0.0	0.0	Std	RI, MS, Std
39	2230	δ-Decalactone	000705-86-2	2.5	Coconut, sweet	109,116.8	353.0	7,873.8	25.5	1,150.2	Std	RI, MS, Std
40	2254	α-Cadinol	000481-34-5	3.3	Herbaceous, woody	1,149.0	1.7	267.7	0.4	0.0	H	RI, MS
41	2263	Ethyl hexadecanoate	000628-97-7	7.8	Mild sweet, waxy	150.0	0.2	0.0	0.0	0.0	D	RI, MS
42	2273	Decanoic acid	000334-48-5	4.1	Fatty, rancid, soapy	7,772.0	12.8	307.2	0.5	0.0	Std	RI, MS, Std
43	2384	1-Hexadecanol	036653-82-4	7.3	Faint, floral, oily, sweet, waxy	2,881.6	2.6	0.0	0.0	0.0	Std	RI, MS, Std
44	2457	δ-Dodecalactone	000713-95-1	3.6	Fruity, sweet	32,178.9	277.6	480.3	4.1	0.0	Std	RI, MS, Std
45	2488	Dodecanoic acid	000143-07-7	4.2	Dry, fatty, metallic, waxy, weak	46,013.8	58.8	0.0	0.0	0.0	Std	RI, MS, Std
46	2587	1-Octadecanol	000112-92-5	8.4	Fatty, oil	2,305.2	2.1	0.0	0.0	0.0	E	RI, MS
47	2681	δ-Tetradecalactone	002721-22-4	4.7	Waxy	8,222.7	13.6	0.0	0.0	0.0	Std	RI, MS, Std
48	2700	Tetradecanoic acid	000544-63-8	5.3	Nearly odorless, very faint, waxy	18,909.4	24.2	0.0	0.0	0.0	Std	RI, MS, Std
49	2901	δ-Hexadecalactone	007370-44-7	5.8	Fruity, sweet, waxy	1,672.0	2.1	0.0	0.0	0.0	Std	RI^6^, MS^7^, Std
50	2910	Hexadecanoic acid	000057-10-3	6.4	Waxy	12,467.7	12.5	0.0	0.0	0.0	Std	RI, MS, Std
Total						429,939.8	1,066.1	51,622.8	99.5	17,291.9		

^1^Log Pow values were obtained from PubChem database (last access date: 27 May 2020).

^2^Odor descriptions were obtained from AroChemBase, except for 2,5-hexanedione, δ-hexalactone, and δ-hexadecalactone. The odor description of three compounds was generated with three researchers using authentic standards.

^3^ ×10^4^.

^4^Quantification: Std, authentic standard; A, toluene; B, 2-hexanone; C, 2-hexanol; D, ethyl decanoate; E, 1-hexadecanol; F, (E,E)-2,4-decadienal; G, 2-undecanone; H, 2-phenoxyethanol.

^5^Identification: *RI* retention index, *MS* mass spectral fragmentation pattern, *Std* authentic standard.

^6^Ref. ^43^.

^7^Ref. ^24^.

**Table 2:** OAV^1^ (>1) of aroma compounds in the EVCO extract obtained by OA-LLE.

Compound	Odor^2^	OAV^1^
δ-Dodecalactone	Fruity, sweet	462.6
δ-Decalactone	Coconut, sweet	176.5
2-Hexanone	Cinnamon, ethereal, fruity	21.1
Ethyl decanoate	Fruity, grape, waxy	19.2
3-Hexanone	Ethereal, fresh, fruity, grape	18.7
δ-Octalactone	Peach, sweet	13.9
(E,Z)-2,4-Decadienal	Deep-fried, fatty, geranium, metallic, tallowy	7.3
Ethyl octanoate	Anise, baked fruity, fatty, floral, fresh, fruity, green, leafy, mentholic, soapy, sweet, waxy	5.0
2-Hexanol	Fatty, fruity, winey	4.3
2-Nonanone	Baked, earthy, fatty, fruity, green, hot milk, soapy	1.4
Acetic acid	Acidic, pungent, sour, vinegar	1.4
1-Dodecanol	Fatty, waxy	1.4
3-Hexanol	Alcoholic, ethereal, medicinal	1.2
2-Undecanol	Fruity	1.1

^1^Odor activity value. OAV was calculated as follows: the concentration of aroma compounds (µg/200 µL) shown in Table 1 was regarded as the concentration of aroma compounds in 5 g of EVCO and was converted to mg/kg by multiplying by 200. The converted concentration of aroma compounds/odor threshold in oil or water from AroChemBase. The underlines indicate that the OAVs were calculated using a threshold in water.

^2^Odor descriptions were obtained from AroChemBase.

**Table 3:** % Area of the (R)- and (S)-δ-lactone enantiomers in the EVCO extract obtained by OA-LLE.

Lactone		% Area
δ-Hexalactone	(R)	89.1 ± 0.1
	(S)	10.9 ± 0.1
δ-Octalactone	(R)	90.8 ± 0.1
	(S)	9.2 ± 0.1
δ-Decalactone	(R)	78.3 ± 0.1
	(S)	21.7 ± 0.1
δ-Dodecalactone	(R)	41.3 ± 0.1
	(S)	58.7 ± 0.1
δ-Tetradecalactone	(R)	72.1 ± 0.4
	(S)	27.9 ± 0.4
δ-Hexadecalactone	(R)	95.9 ± 0.1
	(S)	4.1 ± 0.1

The data represent the mean ± SD (*n* = 3).

### Comparison of extraction efficiency among the methods

**Fig. 4:**
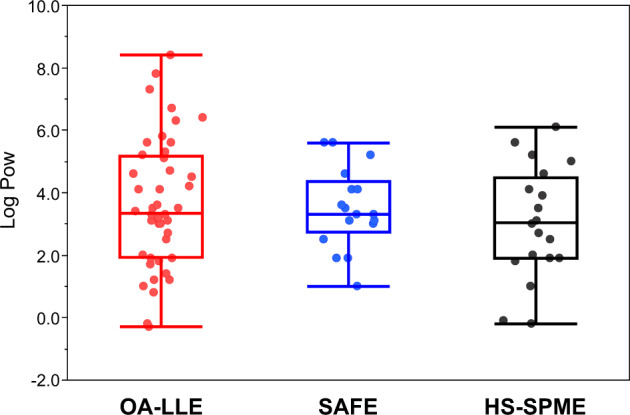
Box plots of the log Pow values for the volatile compounds extracted with each method. The distribution of the data is summarized in box plots and the log Pow values for each aroma compound are plotted. The center line in the box plots is the median, the box denotes the interquartile range (IQR), and whiskers are drawn to the furthest point within 1.5 × IQR from the box. The log Pow values were quoted from PubChem database (last access date: 27 May 2020). The number of volatile compounds of OA-LLE, SAFE, and HS-SPME was 44, 17, and 20, respectively.

The log Pow value of the volatile compounds isolated by each method was plotted and calculated statistically (Fig. 4). A wide range of volatile compounds from log Pow −0.3 (2,5-hexanedione) to log Pow 8.4 (1-octadecanol) were isolated from the triacylglycerols using OA-LLE. Using SAFE, 17 volatile compounds were extracted. The range of log Pow values of the volatile compounds was from 1.0 (δ-hexalactone) to 5.6 (ethyl dodecanoate and 2-tridecanol). Comparing the results of OA-LLE and SAFE, the variances in log Pow values were 4.26 and 1.67, which were statistically different (*p* < 0.05). Using HS-SPME, 20 of the volatile compounds in EVCO were identified with log Pow from −0.2 (acetic acid) to log Pow 6.1 (dodecane). The variance for HS-SPME was 3.11, and the variances of OA-LLE and HS-SPME were not statistically different (*p* = 0.4350). The variances of SAFE and HS-SPME were also not statistically different (*p* = 0.2076).

The total amounts of the aroma compounds in the extracts from OA-LLE and SAFE were 1,066.1 and 99.5 µg/200 µL, respectively. Using OA-LLE, we were able to extract over 10-fold the amount of aroma compounds from EVCO compared with SAFE. δ-Lactones, δ-C_6_ to δ-C_12_, were detected, and the content of these δ-lactones in the extract from SAFE were 16.0 ± 2.2, 40.9 ± 1.8, 25.5 ± 5.6, and 4.1 ± 1.5 µg/200 µL (*n* = 3, mean ± SD), respectively. Compared with the result from OA-LLE, the amount of δ-lactones in the extract from SAFE was much lower, and δ-C_14_ and δ-C_16_ were not detected in the SAFE extract. There were no specific compounds found by SAFE but not by OA-LLE. During the SAFE distillation, EVCO accumulated at the high vacuum stopcock and flash distillation may not have worked well (Supplementary Fig. 1).

The sums of the peak abundances of the GC-MS chromatograms from OA-LLE, SAFE, and HS-SPME were 429,939.8, 51,622.8, and 17,291.9, respectively. The total abundance of HS-SPME was the lowest due to the matrix effect. The specific compounds found by HS-SPME but not by OA-LLE were as follows: 1 alcohol (ethanol), 1 aldehyde (hexanal), 1 ketone (2-heptanone), and 3 hydrocarbons (octane, decane, and dodecane). The extraction efficiency of the volatile compounds from EVCO was dramatically improved using OA-LLE.

### Similarity and intensity test of the aromatic extracts from OA-LLE and SAFE

Similarity and intensity of EVCO aroma perceived by the panelists for the extracts obtained by OA-LLE and SAFE were tested. The mean similarity scores of the aromatic extracts obtained from OA-LLE and SAFE on the smelling strips were 71.4 ± 7.3 and 37.4 ± 6.6 mm (*n* = 5, mean ± standard error (SE)), respectively, and were statistically different (*p* < 0.05). The mean intensity scores of the aromatic extracts obtained by OA-LLE and SAFE on the smelling strips were 67.4 ± 11.9 and 75.0 ± 7.0 mm (*n* = 5, mean ± SE), respectively, and there was no statistical difference (*p* = 0.5650). These results indicate that the OA-LLE extract had a similar aroma composition to the reference sample (EVCO).

## Discussion

**Fig. 5:**
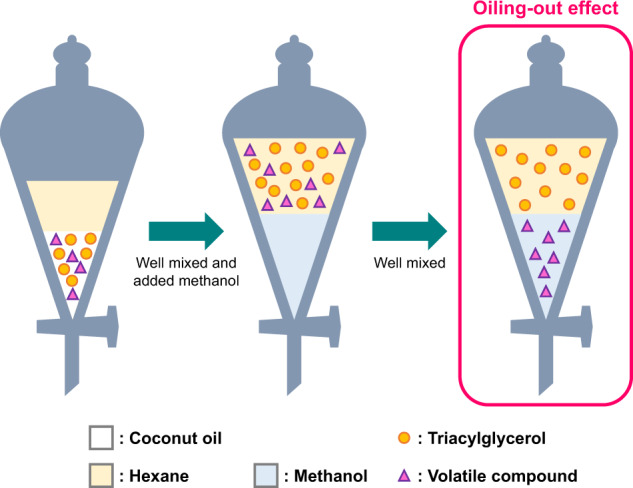
Image of the oiling-out effect in the extraction procedure of EVCO. This liquid-liquid extraction is the first step of OA-LLE. The hexane layer maintained triacylglycerols and pushed out relatively hydrophilic compounds (volatile compounds) into the methanol layer due to the oiling-out effect.

To understand the native profile of volatile compounds in edible oil, we need to overcome the strong matrix effect of oil. Solvent extraction, which is the most widely used method for extracting volatiles, is less susceptible to matrix effects. However, solvent extraction is unsuitable for extracting volatile compounds from oil because volatile compounds and oil are extracted simultaneously. A large amount of oil in the isolate makes it difficult to concentrate volatile compounds^16^. OA-LLE proposed in this paper consists of liquid-liquid extractions, which is less affected by the matrix effect of oil because of solvent-based extraction. The oiling-out effect for EVCO is illustrated in Fig. 5. The oiling-out effect was defined in our previous study^16^.

In brief, we focused on the partition coefficient of oil (middle- and long-chain triacylglycerols) and demonstrated that triacylglycerols affected the distribution of the hexane/methanol bilayer and the equilibrium distribution of aroma compounds when performing liquid-liquid extraction. The partition coefficient of medium- and long-chain triacylglycerols is extremely high (log Pow value >10). According to the PubChem database, the log Pow value of trilaurin, the most abundant triacylglycerol in coconut oil, is 15.6. This phenomenon is similar to the “salting-out effect” of chemicals in aqueous solution by adding sodium chloride. As with the salting-out effect, adding low polarity compounds immiscible with water, such as oil, changes the polarity of the hexane layer and pushes out relatively hydrophilic compounds into the methanol layer. In addition, there is no heating process in OA-LLE. Heating should be avoided to generate artifact compounds during the extraction procedure. In this study, we applied OA-LLE to EVCO as a typical model to propose a fresh approach to extracting volatile compounds from edible oil. As a result, a wide variety and large number of volatile compounds could be isolated from only 5 g of EVCO. In addition, the aroma characteristic of the OA-LLE extract was similar to the reference sample (EVCO).

The main component of the volatiles in EVCO was δ-lactones, which corresponded to the results of previous studies^19–21^. The content of δ-lactones in EVCO was also similar to previous studies, indicating most of the volatile compounds were extracted from EVCO with OA-LLE (Supplementary Table 1)^19,21,23^. In this study, δ-C_16_ was detected in EVCO. Because δ-C_16_ has a high log Pow value, it is considered that these compounds were concentrated in EVCO from coconut meat during the production processes of EVCO. δ-C_16_ contributes to the “buttery” sensation of food, but it may be difficult to evaluate GC-olfactometry and OAV due to the high boiling point^24^.

From our results, it was found that there are many semi-volatile compounds and compounds with high affinity for oil other than δ-lactones in EVCO. Edible oils such as olive oil and coconut oil are consumed as food in general. To understand the aroma and flavor characteristics of edible oils, we should focus on not only volatile compounds but also semi-volatile compounds and compounds with a high affinity for oil. To the best of our knowledge, 23 of the aroma compounds, comprising 2 acids (nonanoic acid and hexadecanoic acid), 13 alcohols (3-penten-2-ol, 3-hexanol, 2-hexanol, 1-hexanol, 2-ethylhexan-1-ol, 2-undecanol, 2-tridecanol, 1-dodecanol, 2-pentadecanol, 2-phenoxyethanol, α-cadinol, 1-hexadecanol, and 1-octadecanol), 2 aldehydes ((E,Z)-2,4-decadienal and (E,E)-2,4-decadienal), 3 hydrocarbons (toluene, 1,2,4-trimethylbenzene, and 4-ethyl-1,2-dimethylbenzene), 2 ketones (3-hexanone and 2,5-hexanedione), and 1 lactone (δ-hexadecalactone), were newly found in coconut oil using OA-LLE^19–21^. The alcohols have a fruity or floral aroma and might contribute to the EVCO aroma. Moreover, 14 of the potential odorants were found by OAV. As for the aroma component in coconut oil, compounds other than δ-lactones have not been focused on so far.

We also revealed the enantiomers of δ-lactone in EVCO, as shown in Fig. 3 and Table 3. Because enantiomers have different aroma characteristics in general, the data on the enantiomeric ratio are important in helping to understand the native aroma^25^. The % area of (R)- and (S)-enantiomers of δ-C_14_ and δ-C_16_ in the coconut product were revealed. The δ-C_6_ to δ-C_12_ of % area were similar to previous studies^26^. Notably, the predominance of (R)-enantiomers decreased with an increasing lactone chain length until δ-C_12_, but increased at δ-C_14_ and δ-C_16_ (Table 3). Lauric acid is the most abundant fatty acid in coconut oil, which might be related to the enantiomeric ratio.

There are four common methods for producing coconut oils, namely expelling, centrifugation, and fermentation with and without heating^27^. In addition, there are many types of processing, such as bleached, resulting in diversified flavors^28^. The application of OA-LLE to EVCO revealed aroma components other than δ-lactones. The minor aroma activity compounds may characterize the variety of coconut oils. Traditionally, coconut oils have been used not only for foods but also for non-food applications^29,30^. Thus, wider research on the volatile composition of coconut oils should be conducted. To identify key aroma compounds in edible oil, applying aroma extract dilution analysis (AEDA) or charm analysis for the extract obtained by OA-LLE would be helpful^31,32^. The semi-volatiles can be subjected to sensory evaluation by adding a standard compound (food-grade is desirable) to the substrate. We can relate aroma compounds in the extract from OA-LLE with sensory attributes by means of pattern recognition techniques that use multivariate statistical analysis such as partial least square (PLS) algorithms as in previous studies^33,34^.

The sensory panelists in this study judged that the SAFE extract from EVCO was not similar to the reference; only 17 volatile compounds were identified. To the best our knowledge, no study has ever applied SAFE to coconut oil. In this experiment, it was difficult to use SAFE to extract the volatile compounds from EVCO as shown in Supplementary Fig. 1. Because the melting point of EVCO is high, it is considered that EVCO solidified because of the heat of vaporization generated by flash distillation. Applying SAFE to other oils that have a high melting point, such as palm oil and lard, might also be difficult. HS-SPME is also affected by the matrix effect of oil, resulting in the lowest total abundance of peak areas. On the other hand, HS-SPME is a solventless method, so there was no solvent peak in the chromatogram, resulting in 20 volatile compounds being extracted. These results indicate that HS-SPME is suitable for analyzing volatile compounds with a low boiling point. Our previous study showed that applying OA-LLE to 5 g of dark chocolate (fat content, 35.3% w/w) was successful in extracting volatile compounds. With an oil content of 100%, EVCO can be regarded as a sample having the strongest matrix effect. Our results here indicate that OA-LLE is a powerful tool for understanding the aroma profiles of edible oils. Indeed, OA-LLE can be applied to other edible oils (olive oil and beef tallow) and was successful in extracting volatile compounds as shown in Supplementary Fig. 2.

Applying OA-LLE to edible oil and oil-enriched food indicates the potential for other studies. Lukić et al. reported on 256 volatile compounds in olive oils using comprehensive two-dimensional gas chromatography with time-of-flight mass spectrometry (GC × GC-TOF-MS) combined with conventional mono-dimensional GC-MS^8^. They extracted volatile compounds in olive oil using HS-SPME. There have been significant developments in analytical instruments over many years, so combining OA-LLE with high-performance analytical instruments may give a deeper insight into the volatile composition of edible oils. The resulting data should help to reveal geographical or variety differences in oil crops as in previous studies^35,36^. Elucidating the mechanisms of triacylglycerol oxidation is also valuable for food science and the food industry. Recently, a method for analyzing triacylglycerol oxidation in detail has been proposed^37^. The data on the native profile of volatile components derived from triacylglycerols may be useful in elucidating the triacylglycerol oxidation mechanisms in edible oils.

For sustainable supply of foods and for reasons of health, controlling food intake and weight gain have become significant issues. Therefore, understanding the mechanisms of oil and fat perception is desired. Lee et al. reported on the involvement of cluster of differentiation 36 (CD36) on the olfactory epithelium in mice in the perception of oleic aldehyde^38^. In addition, the assay system of human CD36 (peptide mimic) was developed to discover additional potential ligands of CD36^39,40^. Applying OA-LLE for edible oils should provide candidates for potential ligands of CD36, and the findings may help to understand the mechanisms of food perception.

In this study, we demonstrated an effective method for extracting volatile compounds from edible oils. As a result, the volatile list, aroma characteristics, data on potential odorants, and the enantiomeric ratio of δ-lactones were obtained from only 5 g of EVCO. Moreover, OA-LLE is simple, rapid, cost-effective, and does not require a heating process. This method is applicable to a wide variety of edible oils and oil- or fat-enriched foods and should provide a new insight into their aroma profiles.

## Methods

### Reagents and samples

All the solvents and reagents used were commercially available. Hexane (96.0+%) and methanol (99.8%) were purchased from Kishida Chemicals Co., Ltd. (Osaka, Japan). Acetic acid (>99.7%), dichloromethane (99.5%), 2,4-decadienal (90.0%+), hexanoic acid (>99.0%), nonanal (>95.0%), nonanoic acid (90.0%+), tetradecanoic acid (>98.0%), and toluene (>99.5%) were purchased from FUJIFILM Wako Pure Chemical Corporation (Osaka, Japan). δ-Hexanolactone (>99.0%), δ-octanolactone (>98.0%), δ-decanolactone (>97.0%), δ-dodecanolactone (>98.0%), δ-tetradecanolactone (>98.0%), ethyl decanoate (>98.0%), 1-hexadecanol (>98.0%), 2-hexanol (>98.0%), 2-hexanone (>98.0%), octanoic acid (>98.0%), 2-phenoxyethanol (>98.5%), and 2-undecanone (>98.0%) were obtained from Tokyo Chemical Industry Co., Ltd. (Tokyo, Japan). Decanoic acid (>99.0%) and dodecanoic acid (>99.0%) were purchased from Sigma-Aldrich (St. Louis, MO, USA). Hexadecanoic acid was acquired from Nacalai Tesque, Inc. (Kyoto, Japan). δ-Hexadecanolactone (>98.0%) was purchased from Soda Aromatic Co., Ltd. (Tokyo, Japan). 2,5-Hexanedione was obtained from Kanto Chemical Co., Inc. (Tokyo, Japan). Alkane mixed solution (C_7_–C_33_, in hexane) was obtained from Hayashi Pure Chemical Ind., Ltd. (Osaka, Japan). Extra virgin coconut oil (EVCO) made in Thailand was purchased from a local market in Japan and stored at −20 °C until used. The EVCO was made using a centrifuge without heating, and the production date was December 2018.

### Extraction of volatile compounds in EVCO

#### Oiling-out assisted liquid-liquid extraction (OA-LLE)

The procedure for extracting volatile compounds in EVCO is shown in Fig. 1. First, 5 g of EVCO and 20 mL of hexane were charged into a separatory funnel and then well mixed. After that, 10 mL of methanol were charged into the separatory funnel and liquid-liquid extraction was performed. The lower methanol layer containing volatiles was recovered, and then the remaining volatile compounds in the upper oil-rich hexane layer were extracted three times with methanol (5.0, 2.5, and 2.5 mL, respectively). Distilled water (531 µL) was added to the combined mixture of methanol layers (26.0 mL, saturated with hexane) until insoluble hexane appeared. After that, the separated hexane layer was removed. Next, distilled water (60.1 mL) was added to the aqueous methanol solution to prepare a 30% aqueous methanol solution. Finally, volatile compounds were extracted four times with dichloromethane (28.9, 14.4, 14.4, and 14.4 mL) from the 30% aqueous methanol layer. The dichloromethane layer was treated with 10 g of anhydrous sodium sulfate to remove the water (overnight at −20 °C). The dehydrated dichloromethane layer was concentrated to 200 µL using a Hempel column (25 × 1.0 cm) with a twisted glass plate under atmospheric pressure (ca. 43 °C). To calculate the recovery of oil, the removed hexane layers were evaporated and dried using a vacuum drying system for 2 h at room temperature. A 2 µL aliquot of the EVCO extract was used for GC-MS analysis. The extraction was performed in triplicate.

#### Solvent assisted flavor evaporation (SAFE)

The method was in accordance with Peres et al. with a minor modification^11^. First, 5 g of EVCO and 25 mL of dichloromethane were placed into a flat-bottom flask with a cap and well mixed. The sample solution was charged into a SAFE apparatus with an oil diffusion pump (10^−3^ Pa)^10^. The gentle distillation of volatiles under a high vacuum distillation system thermostated at 40 °C resulted in the volatiles in dichloromethane. The isolate was dried with 10 g of anhydrous sodium sulfate (overnight at −20 °C) and concentrated to 200 µL using a Hempel column (25 × 1.0 cm) with a twisted glass plate under atmospheric pressure (ca. 43 °C). A 2 µL aliquot of the EVCO extract was used for GC-MS analysis. The extraction was performed in triplicate.

#### Head-space solid-phase micro extraction (HS-SPME)

HS-SPME was conducted using the method described by Santos et al. with a minor modification^21^. In brief, 5 g of EVCO was placed into a 20-mL glass serum vial and 1 µL of cyclohexanol was added as an internal standard. The vial was subsequently screw-capped with a laminated Teflon-silicone disc. HS-SPME was performed with a 50/30 μm divinylbenzene/carboxen/polydimethylsiloxane (DVB/CAR/PDMS) fiber mounted on a SPME manual holder assembly (Supelco, Inc., Bellefonte, PA, USA). The vial containing EVCO was placed in a 40 °C water bath and maintained for 5 min with stirring. The needle of the SPME device was then inserted into the vial through the septum, and the plunger of the SPME apparatus was pushed down to expose the SPME fiber to the vial head space. After 30 min exposure, the fiber was retracted into the needle assembly, removed from the vial, and then introduced into a preheated GC-MS injector port for analyte desorption at 230 °C for 5 min. HS-SPME analysis was performed in triplicate.

### Gas chromatography-mass spectrometry (GC-MS)

The volatile compounds were isolated and identified using a 7890 A GC System gas chromatograph (Agilent Technologies, Inc., Santa Clara, CA, USA) coupled with a 5975 C inert XL MSD mass spectrometer detector (Agilent Technologies, Inc.). The GC-MS system was equipped with a capillary column DB-WAX Ultra Inert (30 m × 0.25 mm i.d., 0.25 µm film thickness; Agilent Technologies, Inc.). The carrier gas was helium (99.995%) and the column head pressure was set at 108 kPa. The oven temperature was set at 30 °C for 2 min, increased to 230 °C at 3 °C min^−1^, and finally maintained at 230 °C for 45 min. The splitless injection mode was at 230 °C. Mass spectra in the electron impact mode (EI) were generated at 70 eV and the MSD transfer line temperature was set to 230 °C. The mass scan range was 30 to 400 *m/z*.

### Identification and quantification of volatiles

To identify the volatile compounds, authentic standards, AromaOffice version 7.0 (Nishikawa Keisoku Co. Ltd., Tokyo, Japan), and AroChemBase version 7.0 (Alpha MOS, Toulouse, France) were used. The volatile compounds were identified based on three criteria: (**1**) by comparing the mass spectra of each compound with the NIST 8.0 mass spectra library, (**2**) by comparing the retention index with the literature data of AroChemBase, and (**3**) by comparing the mass spectra and retention time with an authentic standard. The AroChemBase module consists of a library of chemical compounds with name, formula, CAS number, molecular weight, Kovats retention index, sensory attributes, odor threshold, and related bibliography. For OA-LLE and SAFE, peak area ratios of the analyte to the internal standard (cyclohexanol) were used to calculate each concentration of volatiles in the extracts. In brief, 2 µL of 0.5% (w/v) cyclohexanol in dichloromethane was spiked into each of the extracts and authentic standard solutions (200 µL). Some of the compounds were substituted with a compound that has a similar structure, and the amount was calculated as a semi-quantification.

### Enantioselective GC-MS for δ-lactones

The enantiomers of δ-lactones in EVCO were analyzed using a 7890 A GC System gas chromatograph (Agilent Technologies, Inc.) coupled with a 5975 C inert XL MSD mass spectrometer detector (Agilent Technologies, Inc.) and a Supelco β-DEX™ 225 capillary column (30 m × 0.25 mm, coated with a 0.25 μm layer of liquid phase; Merck KGaA, Germany). The carrier gas was helium (99.995%) and the flow rate was set at 1 mL min^−1^. A 1 µL aliquot of the standard solution and the diluted extract obtained by OA-LLE (50-fold diluted extract for δ-C_6_–C_12_, 5-fold diluted extract for δ-C_14_ and δ-C_16_) was injected. The oven temperature for δ-C_6_–C_12_ was set at 90 °C for 2 min, increased to 160 °C at 2 °C min^−1^, maintained for 70 min, increased to 230 °C at 10 °C min^−1^, and finally maintained at 230 °C for 5 min. The oven temperature for δ-C_14_ and δ-C_16_ was set at 90 °C for 2 min, increased to 160 °C at 2 °C min^−1^, maintained for 120 min, increased to 230 °C at 3 °C min^−1^, and finally maintained at 230 °C for 5 min. The splitless injection mode was at 230 °C. Mass spectra in the electron impact mode (EI) were generated at 70 eV and the MSD transfer line temperature was set to 230 °C. The mass scan range was 30 to 400 *m/z*. Selected ion monitoring (44, 55, 71, and 99 *m/z*) was applied to describe the chromatograms and the enantiometric ratio of δ-lactones. The peaks were identified by comparing the retention times and MS fragment pattern with those of pure racemic standards. The elution order of enantiomers was determined according to the literature^41^. The enantiomeric ratio of δ-lactones contained in the extract from OA-LLE was also calculated from the peak areas obtained by an enantioselective GC-MS. The enantioselective GC-MS analysis was performed in triplicate.

### Log Pow value

The log Pow value of volatile compounds was obtained from PubChem database (last access date: 27 May 2020).

### Odor activity value (OAV)

The OAV of each volatile compound in the extract was calculated by dividing the concentration of a volatile compound by its threshold value in oil or water media. Threshold values were obtained from AroChemBase. Because the aroma extracts were obtained from 5 g of EVCO, the concentration of volatile compounds contained in the OA-LLE extract was converted to mg/kg by multiplying by 200.

### Sensory analysis

The method was in accordance with Selli et al. with a minor modification^42^. In brief, the panelists were instructed to sniff and memorize the aroma of the reference sample, EVCO, and for the extract, to sniff the smelling strip odor and to determine the similarity and intensity of the odors. A smelling strip (Daimonji Paper Co., Ltd., Tokyo, Japan) was used for the representativeness test of the aromatic extract obtained by OA-LLE and SAFE. The extract (15 µL) was applied to the extremity of the smelling strip. After 30 s (the time necessary for solvent evaporation), the smelling strip was given to a panelist. The panelists evaluated the quantity of the odor similarity and intensity of the aromatic extract by comparing with the odor of the reference. For the similarity test, a 100-mm unstructured scale was used anchored with “far from the reference” on the left and “near to the reference” on the right. For the intensity test, a 100-mm unstructured scale was used anchored with “no odor” on the left and “very strong odor” on the right. The position of the sample on the unstructured scale was read as the distance in millimeters from the left anchor. Results were expressed as a mean score of the sensory perceptions of five panelists from our laboratory (one female and four males, 30 to 50 years old). The room temperature was set to 25 °C.

### Statistical analysis

All statistical analyses were carried out using Microsoft Excel version 15.0.4963.1000 or JMP 14.3 provided by SAS Institute Inc. (Cary, NC, USA). Bartlett’s test for homogeneity of variance was carried out to compare the variances of log Pow values. For sensory analysis, the mean scores were compared by the two-sided paired *t*-test, following a test for normality using the Shapiro–Wilk test. A significance level of each analysis was set to *p* < 0.05.

## Supplementary information

Supplementary information

## Data Availability

The authors declare that all data supporting the findings of this study are available within the paper and its supplementary information.
