# Supplementary material for: Oiling-out effect improves the efficiency of extracting aroma compounds from edible oil
Source: NPJ Sci Food. 2020 Nov 4;4:18. doi: 10.1038/s41538-020-00079-8 (PMC7642429; doi:10.1038/s41538-020-00079-8)
Supplement: Supplementary file 1 — Supplementary information [file 41538_2020_79_MOESM1_ESM.pdf]

Supplementary information

Oiling-out effect improves the efficiency of extracting aroma compounds from edible oil

Daisuke Suzuki[1,2*], Yuko Sato[1], Hiroshi Kamasaka[1], Takashi Kuriki[1], Hirotoshi Tamura[2,3]

[1] Institute of Health Sciences, Ezaki Glico Co., Ltd., 4-6-5 Utajima, Nishiyodogawa-ku, Osaka, 555-8502, Japan
[2] The United Graduate School of Agricultural Sciences, Ehime University, 3-5-7 Tarumi, Matsuyama-shi, Ehime, 790-8566, Japan
[3] The Graduate School of Agriculture, Kagawa University, 2393 Ikenobe, Miki-cho, Kagawa, 761-0795, Japan
[*] Corresponding author (daisuke.suzuki@glico.com)

Supplementary Table 1

Supplementary Fig. 1

Supplementary Fig. 2

Supplementary Table 1. The content of δ-lactones in coconut oil (mg/kg)

Lactones	Current	Allen[a]	Santos et al.[b]	Obi et al.[c]
δ-Hexalactone	10.7	27	n.d.	n.d.
δ-Octalactone	34.6	64	69.75–127.71	104.5
δ-Decalactone	70.6	88	<LOD[d]	105.8
δ-Dodecalactone	55.5	65	n.d.	29.9
δ-Tetradecalactone	2.7	20	n.d.	9.12
δ-Hexadecalactone	0.4	n.d.	n.d.	n.d.

For "Current", the concentration of δ-lactones shown in Supplementary Table 1 was regarded as the concentration of aroma compounds in 5 g of extra virgin coconut oil (EVCO) and was converted to mg/kg by multiplying by 200. Although the current result shows the actual δ-lactones content in the oiling-out assisted liquid-liquid extraction (OA-LLE) extract, the concentration is similar to that of the previous studies. Most of the δ-lactones were isolated from triacylglycerols by OA-LLE.

[a] Ref. 19, Allen obtained δ-lactones from coconut oil using vacuum distillation.

[b] Ref. 21, Santos et al. obtained δ-lactones from coconut oil using HS-SPME.

[c] Ref. 23, Obi et al. obtained δ-lactones from coconut oil using solvent extraction.

[d] <LOD, limit of detection

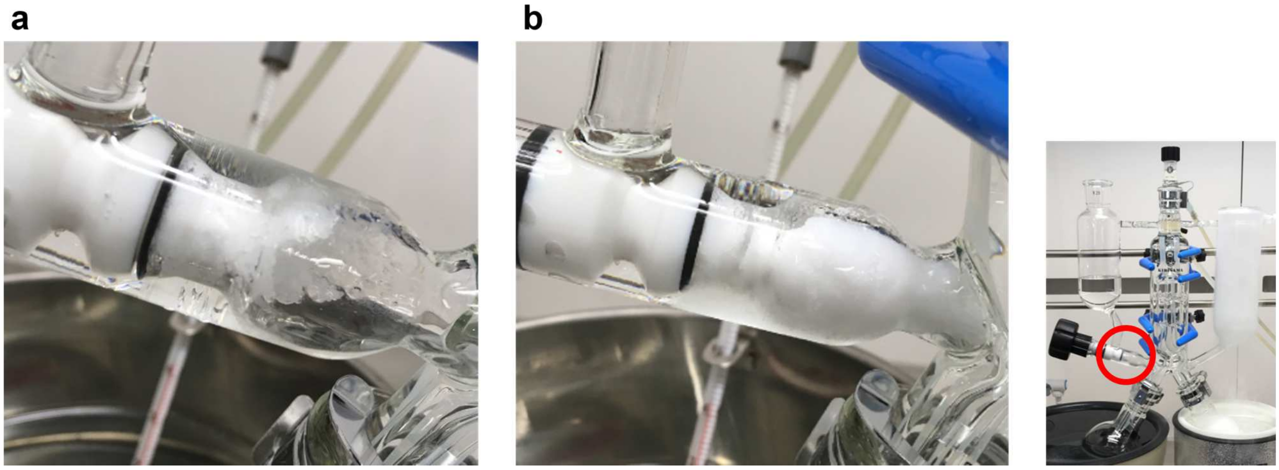

Supplementary Fig. 1 Application of solvent assisted flavor evaporation (SAFE) to EVCO
Despite warming surfaces of the sample inlet (red circle) with a heat gun, EVCO was gradually accumulated at the sample inlet during SAFE extraction (**a**). After SAFE extraction, EVCO was solidified at the sample inlet (**b**).

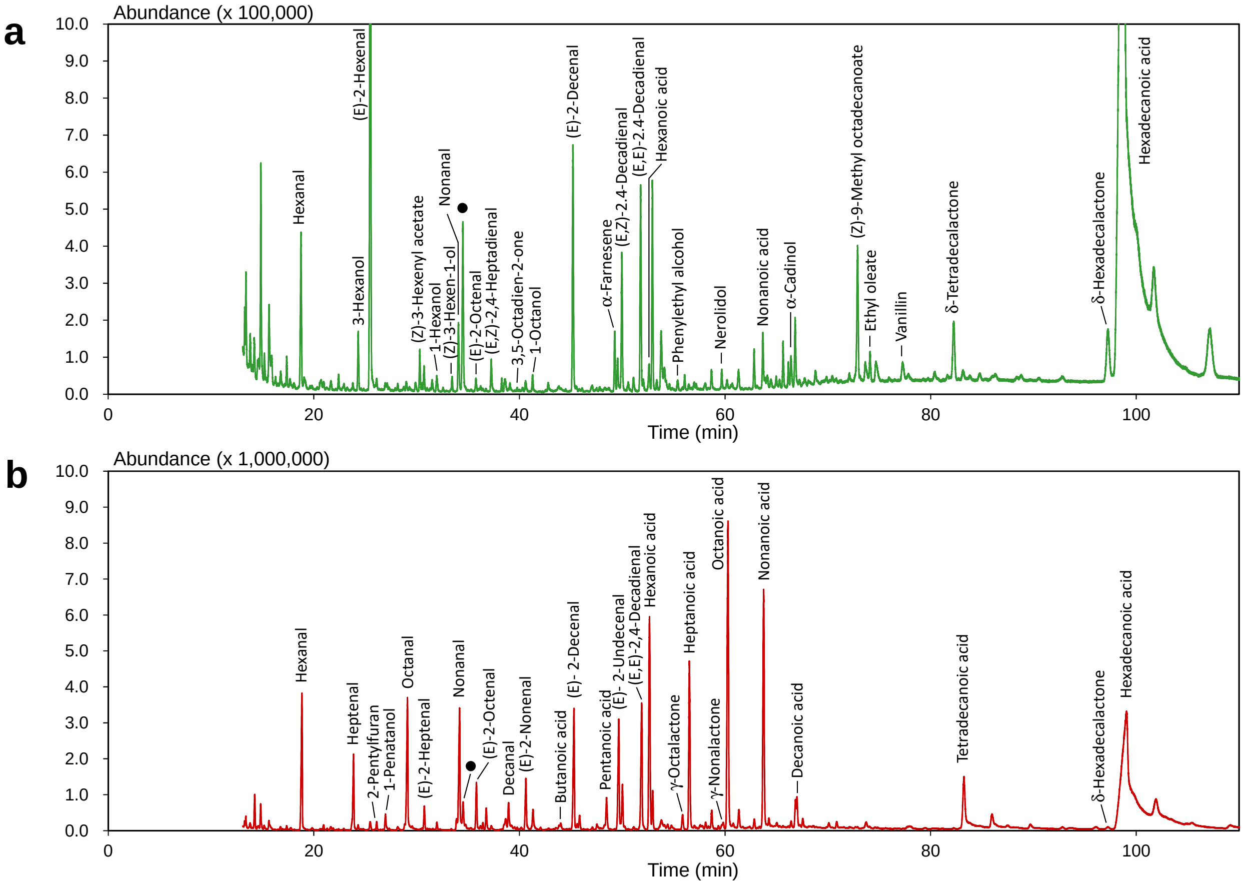

Supplementary Fig. 2 GC-MS chromatograms of the extracts from edible oils using OA-LLE
Each aroma extract was obtained from 5 g of olive oil (Toscana, Italy) (**a**) and beef tallow (**b**). The extraction procedure and analytical method were the same as for EVCO. Identification of volatiles was performed using the NIST'08 mass spectral database and retention index database registered in AroChemBase. Filled black circles (•) indicate the peak of internal standard (0.005% (w/v) cyclohexanol).
